# A87 A SURVEY-BASED ASSESSMENT OF ENDOSCOPY CURRICULUM AND EDUCATION IN CANADIAN TRAINING PROGRAMS

**DOI:** 10.1093/jcag/gwad061.087

**Published:** 2024-02-14

**Authors:** M Gozdzik, A Kundra, R Snelgrove, K Kroeker

**Affiliations:** Gastroenterology, University of Alberta, Edmonton, AB, Canada; University of Virginia, Charlottesville, VA; Gastroenterology, University of Alberta, Edmonton, AB, Canada; Gastroenterology, University of Alberta, Edmonton, AB, Canada

## Abstract

**Background:**

Endoscopic training within Canada occurs in programs accredited by the Royal College of Physicians and Surgeons of Canada (RCPSC). Beyond national accreditation, there are no standardized endoscopic training curriculums and each program is responsible for ensuring its trainees achieve competency. There is debate in the literature regarding the minimum number of endoscopic procedures and the role of objective assessment tools in the assessment of trainee competence with no specific recommendations for these by the RCPSC.

**Aims:**

The goal of our study was to characterize endoscopic training curriculums within Canadian training programs while determining the usage and perceived importance of procedure volume tracking and objective assessment tools.

**Methods:**

Online surveys were sent to program directors (PD) and trainees within Adult Gastroenterology, Pediatric Gastroenterology, General Surgery and Colorectal Surgery programs within Canada. Surveys contained questions meant to characterize endoscopy training curriculums and perceived effectiveness of the curriculum while highlighting the factors important for feedback and competency assessment.

**Results:**

Survey responses were obtained from 20 PDs and 83 trainees. Of all programs surveyed 53% (44/83) of trainees reported that they had a formal endoscopy curriculum compared with 75% (15/20) of PDs. PDs assessed endoscopic competence predominantly by EPAs (95%) and verbal feedback (65%) with minimal usage of objective tools (30%). Trainees reported fewer objective tool usage at 2.4% (2/83). When assessed on a 5-point Likert scale, both PDs and trainees felt that verbal feedback was the most important for assessment of competence (3.58 and 4.39 respectively). Seventy-seven percent of trainees report using a procedure log and when asked about procedure volume 37% of trainees expected to complete ampersand:003C250 colonoscopies by the end of their training. Eighty-three percent of PDs felt that all their trainees achieved endoscopic competency with only 50% of adult GI PDs reporting the same. Most PDs and trainees felt that national recommendations for objective tool usage (65% and 57% respectively) and minimum procedure volume (65% and 63% respectively) would be helpful to standardize endoscopic training. Additional results are reported in Figure 1.

**Conclusions:**

Most training programs reported a standardized curriculum with a majority of trainees tracking their procedure volume, but a minority of programs incorporated objective tools. A significant portion of trainees expected to complete ampersand:003C250 colonoscopies by the end of their training and not all PDs felt that all of their trainees reach endoscopic competence. The majority of trainees and PDs felt national recommendations for procedure tracking and objective tools would be helpful, suggesting the need for more formalized endoscopic curriculums in Canada.

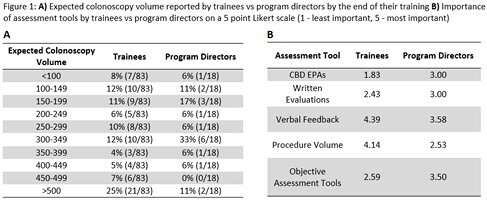

**Funding Agencies:**

None

